# Quercetin as a nephroprotector after warm ischemia: histomorphometric evaluation in a rodent model

**DOI:** 10.1590/S1677-5538.IBJU.2020.0358

**Published:** 2021-02-28

**Authors:** Gabriela F. Buys Gonçalves, Maria Eduarda M. Silva, Francisco J. B. Sampaio, Marco A. Pereira-Sampaio, Diogo Benchimol de Souza

**Affiliations:** 1 Universidade do Estado do Rio de Janeiro - UERJ Unidade de Pesquisa Urogenital Rio de JaneiroRJ Brasil Unidade de Pesquisa Urogenital, Universidade do Estado do Rio de Janeiro - UERJ, Rio de Janeiro, RJ, Brasil;; 2 Fundação Educacional Serra dos Órgãos Departamento de Medicina Veterinária TeresópolisRJ Brasil Departamento de Medicina Veterinária, Fundação Educacional Serra dos Órgãos, Teresópolis, RJ, Brasil;; 3 Universidade Federal Fluminense - UFF Departamento de Morfologia NiteróiRJ Brasil Departamento de Morfologia, Universidade Federal Fluminense - UFF, Niterói, RJ, Brasil

**Keywords:** Warm Ischemia, Quercetin, Nephrectomy

## Abstract

**Purpose:**

To quantitatively evaluate the possible long-term protective effects of quercetin during renal warm ischemia.

**Materials and Methods:**

Male rats were allocated into 4 groups: sham (S), sham quercetin (SQ), ischemia (I), and ischemia quercetin (IQ). Groups SQ and IQ received quercetin (50mg/kg) before and after surgery. Groups I and IQ had their left renal vessels clamped for 60 minutes. All animals were euthanized four weeks after the procedure, and serum urea and creatinine levels were measured. Renal weight and volume, cortex-non-cortex area ratio (C-NC), cortical volume (CV), glomerular volumetric density (Vv[glom]), volume-weighted glomerular volume (VWGV) and number of glomeruli per kidney (N[glom]) were evaluated by stereological methods. Results were considered statistically significant when p <0.05.

**Results:**

Serum urea levels in group I increased by 10.4% in relation to group S, but no differences were observed among the other groups. The C-NC of group I was lower than those of all other groups, and group IQ had similar results to sham groups. The Vv[glom] and N[glom] of group I were lower than those of group S (33.7% and 28.3%, respectively) and group IQ had no significant difference compared to the S group.

**Conclusions:**

Quercetin was effective as a nephroprotective agent in preventing the glomerular loss observed when the kidney was subjected to warm ischemia. This suggests that this flavonoid may be used preventively in kidney surgery, when warm ischemia is necessary, such as partial nephrectomy.

## INTRODUCTION

Partial nephrectomy has been considered the gold standard treatment for localized small renal masses (1, 2) and has been increasingly performed due to the evolution of diagnostic techniques (3). Despite the constant research and development into new surgical techniques, which include cold ischemia and zero ischemia (4, 5), renal warm ischemia is still mostly used to obtain a bloodless operative field during partial nephrectomy (6).

In contrast, renal ischemia during nephron sparing surgery is associated with postoperative glomerular loss and functional decline of the remaining parenchyma (7, 8), which may lead to renal failure. Therefore, drugs that may have protective effects on the kidney during the preoperative, perioperative, and postoperative periods are warranted (9, 10).

Quercetin has shown positive results under different experimental conditions, mainly attributed to its antioxidant and anti-inflammatory properties (11-14). This flavonoid has also been shown to be promising when associated with warm ischemia in different organs and tissues (15-17) and, more specifically, in the kidney (18-20). However, to the best of our knowledge, quercetin has not yet been investigated for the prevention of glomerular loss after warm ischemia.

The hypothesis of the present study is that quercetin may ameliorate the renal damage caused by ischemia-reperfusion. The objective is to quantitatively evaluate the possible long-term protective effects of quercetin during renal warm ischemia in a rodent model.

## MATERIALS AND METHODS

All experiments were performed in accordance with national and international laws for the scientific use of animals, and this project was formally approved by the Institutional Ethics Committee for Animal Experimentation (CEUA 040/2017).

Forty male Wistar rats aged 9 weeks were used. The animals were randomly allocated into 4 groups: Sham (S) - group submitted to laparotomy and dissection of the renal pedicle (n=10); Sham Quercetin (SQ) - group treated with quercetin and subjected to the same procedures as group Sham (n=10); Ischemia (I) - group submitted to renal warm ischemia (n=10); Ischemia Quercetin (IQ) - group treated with quercetin and submitted to renal warm ischemia (n=10). Groups SQ and IQ received 50mg/kg quercetin (Quercetin, New Roots Herbal, Vaudreuil-Dorion, Canada) by gavage 3 days before and 3 days after surgery, and one intraperitoneal administration 60 minutes before the surgical procedure, while untreated groups (S and I) received saline (instead of quercetin) using the same protocols.

All rats were anesthetized via intramuscular ketamine (Cetamin, Syntec, Santana de Parnaíba, Brazil, 100mg/kg) and xylazine (Xilazin, Syntec, 20mg/kg). Under aseptic technique, a ventral midline incision was used to expose the abdominal viscera, which was displaced to expose the left kidney. The left renal artery and vein were isolated by blunt dissection. In animals of groups I and IQ, the renal vessels were clamped for 60 minutes, while in groups S and SQ the pedicle was dissected, but no ischemia was induced. All groups remained anesthetized for 60 minutes, during which the abdominal viscera were replaced into the abdomen, and the incision was covered with moistened gauze. At the end of this period, vascular clamps were removed and reperfusion was observed (in groups I and IQ), and the abdominal cavity was closed in a standard fashion.

The animals were euthanized 4 weeks after surgery by anesthetic overdose (Isoflurane, BioChimico, Rio de Janeiro, Brazil). Immediately after death, a blood sample was collected by cardiac puncture. This sample was used for urea and creatinine serum levels analysis by the automated enzymatic method.

The left kidneys were collected and fixed in 4% phosphate-buffered formaldehyde. The kidneys were weighed, and the renal volume was measured using Scherle's method (21). The cortex-non-cortex area ratio (C-NC) was calculated using the Cavalieri method (22-24). The cortical volume (CV) was calculated by multiplying the renal volume by C-NC (24).

Randomly collected samples from all left kidneys were processed for paraffin embedding, sectioned at 5µm thickness, and stained with hematoxylin and eosin. Histological analysis was performed blindly using 25 histological fields, obtained from five different sections of the renal cortex from each kidney. Glomerular volumetric density (Vv[glom]), which indicates the proportional volume occupied by the glomeruli in the cortex, was estimated by the point-counting method (22-24). The volume-weighted glomerular volume (VWGV) was estimated using the point-sampled intercepts method (22-24). The estimation of the total number of glomeruli per kidney (N[glom]) was calculated using the formula: CVxVv[glom]/VWGV (24).

The results were compared by one-way ANOVA with Bonferroni's post-hoc test. Analyses were performed using GraphPad Prism 5.0 (GraphPad Software, San Diego, USA). All results were considered significant when p <0.05. All numerical data were presented as mean±standard deviation.

Additionally, an experienced veterinary pathologist (unaware of the treatment conditions) examined all histological sections and described the histopathological changes. The observed alterations were then reported per group.

## RESULTS

The serum urea level in group I was 10.4% higher than that in group S. No difference in serum urea levels were observed between group IQ and other groups. Also, no difference was observed in serum creatinine analysis between all the groups.

The C-NC of group I was decreased by 9.3% when compared to group S. Quercetin treatment in group IQ restored normal values for this parameter. The C-NC of group IQ was 5.9% higher than that of group I, and similar values to groups S and SQ.

The Vv[glom] of group I was 32.7% lower than that of group S and 34.2% reduced in comparison to group SQ. Meanwhile, group IQ increased by 33.7% in comparison to the I group, and a decrease of 12.0% in comparison to SQ. Treatment with quercetin in animals subjected to renal ischemia (group IQ) prevented the reduction of Vv[glom] observed in non-treated animals.

The ischemic kidneys (group I) presented a reduced N[glom] in comparison to groups S and SQ (26.0% and 33.0%, respectively). Treatment with quercetin in group IQ prevented this glomeruli loss, as this group showed no difference with groups S and SQ, but 28.3% increased N[glom] in comparison to group I.

**Figure 1:**
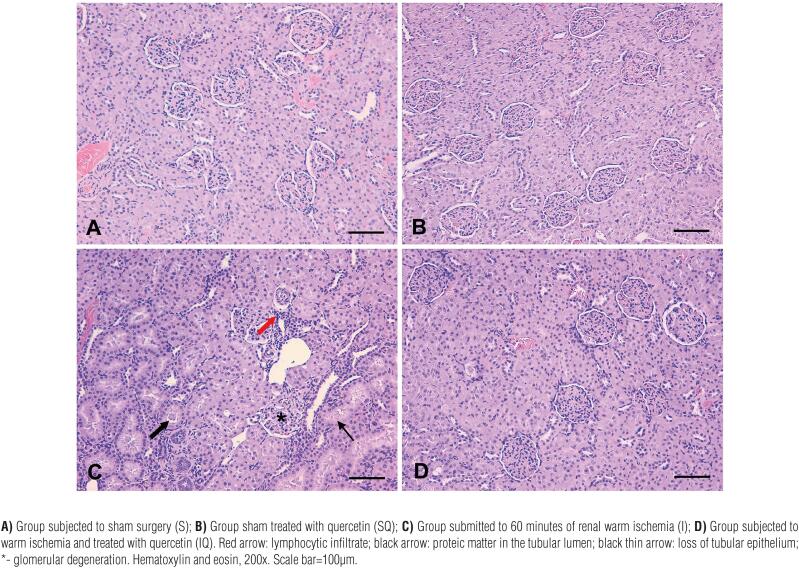
Photomicrographs of the renal cortex from the experimental groups.

**Figure 2:**
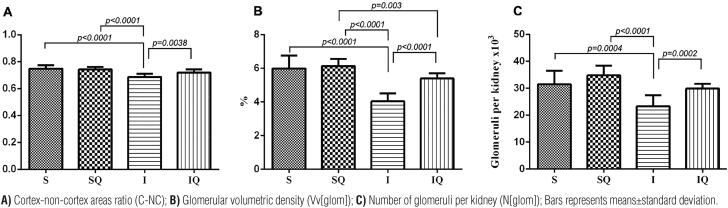
Quantitative results of kidneys from group submitted to sham surgery (S), group sham treated with quercetin (SQ), group submitted to 60 minutes of renal warm ischemia (I), and group subjected to warm ischemia and treated with quercetin (IQ).

No difference was noted in kidney weight, kidney volume, cortical volume, and VWGV among the groups. All data are presented in Table-1 and are represented in Figures 1 and 2.

**Table 1:** Kidney functional and morphological data of rats subjected to sham surgery or to left renal warm ischemia with or without quercetin treatment.

	S (n=10)	SQ (n=10)	I (n=10)	IQ (n=10)	p value
Urea (mg/dL)	41.5±1.7	41.0±1.6	45.8±3.7 a, b	42.7±2.4	0.0013
Creatinine (mg/dL)	0.44±0.01	0.47±0.04	0.47±0.03	0.46±0.05	0.2744
Kidney weight (g)	1.08±0.15	1.24±0.15	1.23±0.21	1.19±0.11	0.1488
Kidney volume (mL)	1.06±0.15	1.19±0.15	1.18±0.18	1.17±0.11	0.1494
Cortex-non-cortex areas ratio	0.74±0.02	0.74±0.02	0.68±0.02 a, b	0.72±0.02 c	<0.0001
Cortical volume (mL)	0.79±0.09	0.88±0.11	0.82±0.11	0.84±0.09	0.2879
Vv[glom] (%)	5.99±0.77	6.14±0.43	4.03±0.46 a, b	5.40±0.31^b^, ^c^	<0.0001
VWGV (×105µm3)	15.09±1.79	15.50±1.61	14.36±2.08	15.23±1.31	0.5143
Number of glomeruli per kidney (×103)	31.50±4.90	34.8±3.62	23.30±4.10 a, b	29.9±1.76 c	<0.0001

**Vv[glom]** = Glomerular volumetric density; **VWGV** = Volume-weighted glomerular volume; **S** = Group subjected to sham surgery; **I** = Group subjected to 60 minutes of renal warm ischemia; **SQ** = Group sham treated with quercetin; **IQ** = Group subjected to warm ischemia and treated with quercetin.

a different from group S

b different from group SQ

c different from group I

Data expressed as mean±standard deviation.

Histopathological analysis revealed discrete tubular and medullary congestion in some animals of groups S and SQ, but the cortical architecture was preserved. On the other hand, many focuses of parenchymal damage were observed in the kidneys of group I. This included amorphous material in the tubular lumen, amorphous eosinophilic material in the medullary region, glomerular degeneration, mononuclear infiltration, apparent loss of glomeruli, and loss of cortical architecture. These pathological findings were minimal in kidneys subjected to ischemia under quercetin treatment (group IQ).

## DISCUSSION

Despite great efforts to avoid warm ischemia during partial nephrectomy, with off-clamp and cold ischemia techniques (4, 5), warm ischemia is still necessary during most nephron sparing surgeries (1, 2, 6), and its harmful and permanent consequences for renal function and the morphology of the remaining parenchyma (7, 8) are caused by the high production of reactive oxygen species and consequent lipid peroxidation (25, 26). Given this, the use of antioxidant drugs as possible nephron protectors is plausible (9-11, 18-20) as well as in other organs and tissues (12-17) associated with ischemia/reperfusion (9, 10, 15-20, 26).

Quercetin is a flavonoid that has proven to be effective in combating the production of reactive oxygen species and has positively modified the values of biochemical markers of homeostatic functions of organs and tissues (12-17), including kidneys in different situations (11) such as warm ischemia (18-20). Our quantitative results corroborate that the use of quercetin is beneficial in warm renal ischemia. It is thought that the protective results observed in animals receiving quercetin were a consequence of the reduced oxidative damage in renal tissue. However, oxidative stress has not been directly evaluated in the present study, as in others (27), and future studies should clarify the mechanisms of action. The use of this flavonoid was effective in preventing glomerular loss (N[glom]), which occurred only in animals subjected to renal ischemia without quercetin treatment.

Although the focus of the present study was histo-morphometric evaluation, the serum urea and creatinine levels were also analyzed, which are the most commonly used biochemical markers of renal function (28). Serum urea increased only in the ischemia group, reinforcing that treatment with quercetin prevented renal dysfunction as indicated by this marker. This is in accordance with previously data reported in previous studies (18-20).

Nephron sparing surgery has, as the name indicates, the objective of removing small kidney masses while preserving the maximum number of nephrons to preserve the kidney's function in the long term (1, 2, 6-8). Therefore, quantification of the absolute number of glomeruli, which is a result that approximates the number of nephrons present in the parenchyma (10, 22-24), provides a definitive result that indicates if the partial nephrectomy reaches its goal. To the best of our knowledge, this study is the first to report the preventive effects of quercetin on glomerular loss caused by warm renal ischemia.

The rodent model is widely used in studies focused on the effects of renal ischemia/reperfusion (9, 18-20, 22), and the morphometric changes caused by this procedure have already been proven (22). Our results are consistent with a previous report of 60-minute warm renal ischemia in this model (22), with a reduction in C-NC, Vv[glom], and N[glom] in the ischemia group compared to the sham group.

Sham animals treated with quercetin presented no clinical, biochemical, or morphometric quantitative renal modifications, which demonstrates that quercetin has no side effects on the kidney, which is in accordance with previous studies (18-20, 26, 29). More importantly, our results confirm that this flavonoid prevents glomerular loss caused by ischemia/reperfusion, since the values of Vv[glom] and N[glom] of the Ischemia group treated with quercetin were similar to those of the sham group.

Previously published studies showed the protective effects of quercetin in a short-term period, as the analysis of these studies was performed one day after renal warm ischemia (18-20). The present study added information on this topic, analyzing the kidneys 4 weeks after the ischemic injury, thus demonstrating that the protective effects of quercetin are permanent.

From a translational perspective, these results suggest that the patient undergoing partial nephrectomy could be treated with quercetin during pre, trans, and postoperative periods with satisfactory prevention of long-term renal dysfunction and chronic kidney disease. Quercetin is commonly found in capsules, and sold as a natural supplement in many countries. It has not been reviewed by the FDA to determine whether it is safe or effective, but in Canada it is officially approved as an antioxidant and capillary/blood vessel protectant (30). Prior to clinical use, it should be investigated in controlled trials, which, taking into consideration the positive findings of this experimental study, are highly warranted.

Nevertheless, it should be noted that the present study had some limitations. The pathophysiological responses to ischemic injury differ among species. Although the rat model of renal warm ischemia is widely used, this is still an experimental setting, with several differences to the clinical setting. Additionally, the evaluation of renal clearance and other renal serum biomarkers would be desirable for a more sensitive functional analysis. Future clinical studies investigating the use of quercetin as a nephroprotective drug in patients who underwent partial nephrectomy with warm ischemia are warranted.

Thus, it is concluded that quercetin was effective as a nephroprotective agent, reducing the histopathological modifications, preventing glomerular loss, and maintaining normal urea serum levels after warm ischemia. These results suggest that this flavonoid may improve long-term renal function in patients who underwent partial nephrectomy with warm ischemia.
